# Ovarian Vein Thrombosis: An Unusual Cause of Abdominal Pain in Breast Cancer

**DOI:** 10.7759/cureus.37363

**Published:** 2023-04-10

**Authors:** Madiha Ahmed, Taaha Mendha, Van Do, Steve Carlan, Mario Madruga

**Affiliations:** 1 Internal Medicine, Orlando Regional Medical Center, Orlando, USA; 2 Internal Medicine, University of Miami, Miller School of Medicine, Miami, USA; 3 Obstetrics, Orlando Regional Medical Center, Orlando, USA

**Keywords:** breast cancer, ovarian vein thrombosis, thromboembolic disease, therapeutic anticoagulation, atypical abdominal pain, breast cancer treatment, idiopathic ovarian vein thrombosis

## Abstract

Ovarian vein thrombosis (OVT) is a rare but potentially life-threatening complication that is usually seen in the intrapartum or postpartum period but can also be seen in patients with risk factors for venous thromboembolism. When symptomatic, it usually presents with abdominal pain and other vague constitutional symptoms, hence it is important for healthcare professionals to be aware of this condition when evaluating patients with risk factors. We present a rare case of OVT in a patient with breast cancer. Due to a lack of clear guidelines regarding the treatment and duration of treatment in non-pregnancy-related OVT, we followed the guidelines for the treatment of venous thromboembolism and started the patient on rivaroxaban for a three-month duration with close outpatient follow-up.

## Introduction

Abdominal pain is one of the most common chief complaints that patients present with in the clinic or emergency department [1]. The underlying pathology of abdominal pain is vast, and identification of the cause and ruling out life-threatening pathology is essential. The differential becomes broader in a female patient, as it encompasses gynecological conditions such as ovarian torsion, ovarian cyst, and ovarian vein thrombosis (OVT). OVT is a clot in the ovarian vein, which drains the ovary. On the left side, the ovarian vein drains into the left renal (kidney) vein, and on the right side, the ovarian vein drains into the inferior vena cava. Since OVT can be a potentially life-threatening condition, it is imperative that OVT is diagnosed and treated early. This timely diagnosis and treatment can help avoid complications, some of which include sepsis and pulmonary embolism [2]. Here, we describe a rare case of OVT that presented as abdominal pain.

## Case presentation

**Figure 1:**
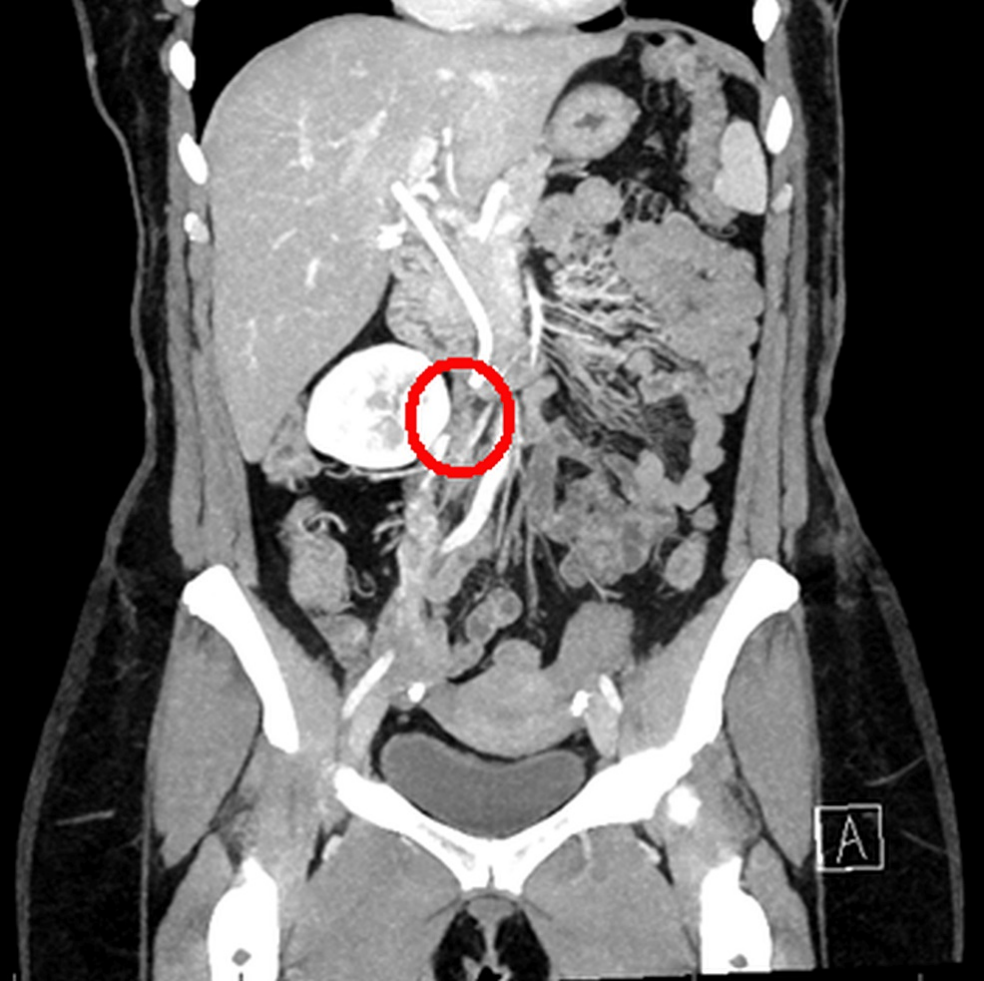
CT scan of the abdomen and pelvis with intravenous contrast showing right ovarian vein thrombosis in the red circle.

A 46-year-old female presented to the emergency department in January 2023 with a complaint of worsening right lower quadrant abdominal pain, which started two days ago. Her past medical history was significant for stage IIb left breast invasive ductal adenocarcinoma and sphincter of Oddi dysfunction status post multiple endoscopic retrograde cholangiopancreatographies (ERCP) with common bile duct dilation and common bile duct stent placement three days prior to presentation to the emergency department. She underwent a modified radical mastectomy of the left breast and chemotherapy in July 2022 and completed adjuvant radiation therapy in November 2022. Thereafter, she was started on abemaciclib, a CDK4/6 inhibitor, and anastrozole, an aromatase inhibitor. She had no prior history of a thromboembolic event and was not on oral contraceptives. Upon examination, she was afebrile and hemodynamically stable. Her physical exam demonstrated epigastric and right lower quadrant tenderness to deep palpation without any guarding, palpable masses, or organomegaly. There was no leukocytosis seen in her laboratory investigations. Computed tomography (CT) scan of the abdomen and pelvis with intravenous (IV) contrast showed that the stent in the common bile duct was in a satisfactory position and demonstrated thrombosis of the right ovarian vein, establishing the diagnosis of OVT (Figure 1).

Given that the abdominal pain was in the setting of a recent history of ERCP and stent placement, she was also evaluated by the gastroenterology team who deemed the pain to be secondary right OVT rather than gastrointestinal in origin. The patient was started on a direct oral anticoagulant, rivaroxaban, for at least a three-month duration of anticoagulation with close outpatient follow-up.

## Discussion

OVT is a rare but potentially life-threatening complication that is usually seen in the intrapartum or postpartum period [3]. Other risk factors for OVT include estrogen-containing hormone treatment, malignancy, recent surgery, and pelvic infections [2]. Most of the data in terms of the incidence and reported cases are in the context of the postpartum patient population. The incidence of OVT is 0.18% post-normal vaginal delivery and 2% post-cesarean section. It has been proposed that the incidence increases with twin pregnancies [1]. However, in our case, the patient was not postpartum. Our patient had a history of cancer and was on abemaciclib; these are two independent risk factors for venous thrombosis. The correlation between cancer and thrombosis has long been established. Several studies have been performed to investigate the potential mechanism of cancer-related hypercoagulability, one of them being the activation of procoagulant molecules by tumor cells leading to an increased risk of venous thromboembolism (VTE) [4]. However, there is very scarce literature on cancers causing OVT, and even when they do, the most common malignancies associated with the development of OVT are genitourinary cancers such as ovarian cancer, uterine, or gastrointestinal. Breast, lung, or hematologic cancers have been less commonly reported [5,6]. The use of CDK4/6 inhibitors such as abemaciclib has also been associated with higher incidences of VTE. A multicenter observational study published by Watson et al. concluded that abemaciclib was associated with a higher risk of VTE and that patients developing thrombosis on abemaciclib had a significantly higher risk of death [7]. There are few reported cases of OVT in patients on abemaciclib despite documented increased risk of VTEs. Hence, there is a need for reports evaluating the role of thromboprophylaxis in patients receiving abemaciclib.

OVT can pose several complications, which include pulmonary embolism, progression of thrombus progression into the inferior vena cava (for right OVT) or into the left renal vein (for left OVT), pelvic congestion syndrome, and recurrent VTE. Ovarian infarction, obstruction of the right ureter causing hydronephrosis, renal failure, and septic pelvic thrombophlebitis, though rare, have also been reported in the literature [2]. Hence, it is very important that a prompt diagnosis is made and appropriate treatment is immediately initiated.

OVT can present with vague, non-specific symptoms such as in our patient. In a multicenter study including 74 women with OVT of various etiologies, 89% had acute onset of symptoms, 7% had chronic symptoms, and 4% were asymptomatic [8]. Alsheef et al. published a case series of retrospective data collection of patients diagnosed with OVT over an 11-year period [9]. The study concluded that the most common presentation was abdominal pain, followed by fever and vomiting. Given that OVT presents with vague symptoms, with the lack of any classic pathognomonic signs, it is important for healthcare professionals to be aware of this condition and to have it on their differential when evaluating patients with risk factors.

If the index of suspicion is high, the right imaging modality needs to be ordered to make the diagnosis. Among published cases of OVT in the literature, several cases used Doppler ultrasound as the initial imaging modality, followed by a CT scan of the pelvis, which confirmed the diagnosis. Doppler ultrasound is non-invasive, inexpensive, and readily available; however, its sensitivity is approximately 50%. It is highly user-dependent and can be limited by body habitus, overlying structures, and bowel gas patterns [10]. A CT venogram or CT scan of the abdomen and pelvis with contrast is highly sensitive and specific for the diagnosis and is time and cost-effective [9]. A retrospective study reported that magnetic resonance angiography (MRA) performed better than CT, making it the gold standard for the diagnosis of OVT [11]. However, MRA is expensive and not widely available. In all of the reported cases, almost all the patients were successfully diagnosed with a CT scan of the abdomen and pelvis with IV contrast, making it the imaging of choice for the diagnosis of OVT.

There are no clearly delineated guidelines regarding the treatment of non-pregnancy-related OVT, the choice of anticoagulant, and the optimal duration of anticoagulation. The guidelines of the British Committee for Standards in Haematology recommend an anticoagulant treatment duration of three to six months for women with postpartum OVT [12]. For non-pregnancy-related OVT, some experts suggest following the general guidelines for the treatment of lower extremity deep vein thrombosis and prescribing anticoagulation for three months in patients with transient risk factors (e.g., infection and surgery) and longer duration in patients with persistent risk factors (e.g., cancer and severe thrombophilia). There is a need for studies to be conducted that evaluate the safety and efficacy of direct oral anticoagulants in the management of OVT, the optimal duration of therapy, and the need for follow-up imaging.

## Conclusions

OVT is a rare disorder, with most of the available literature being on pregnant or postpartum patients. Epidemiological data on other types of OVT and precise estimates of its incidence and prevalence in the general population are scarce. Our case highlights that the presence of any risk factors for VTE should heighten one’s clinical suspicion for OVT in the female patient presenting with abdominal and/or flank pain. An awareness of this condition is important so that necessary imaging can be promptly ordered to establish the diagnosis. Our case report aims to increase awareness within the medical community to reduce diagnostic delays, initiate prompt treatment to avoid complications, and improve patient outcomes.
